# Sex Differences in Substrate Oxidation During Incremental Exercise: An Exploratory Study on the Role of Body Composition

**DOI:** 10.3390/sports14100444

**Published:** 2026-10-09

**Authors:** Carlos Abraham Herrera-Amante, Rodrigo Yáñez-Sepúlveda, José Raúl Hoyos-Flores, Exal Garcia-Carrillo, José Adrián Montenegro-Espinosa, Fiorella Quiroz-Cárdenas, Boryi A. Becerra-Patiño, José Francisco López-Gil

**Affiliations:** 1 Laboratorio de Evaluación y Cuidado del Estado Nutricio, División de Ciencias de la Salud, Centro Universitario de Tonalá, Universidad de Guadalajara, Tonalá 45425, Jalisco, Mexico; carlos.amante@academicos.udg.mx; 2 Red Iberoamericana de Investigadores en Antropometría Aplicada, 04120 Almería, Spain; jose.hoyosfl@uanl.edu.mx; 3 Faculty of Education and Humanities, School of Sport Sciences, Universidad Andres Bello, Viña del Mar 2520000, Chile; rodrigo.yanez.s@unab.cl; 4 Department of Sport Sciences, Faculty of Sport and Health Sciences, Fit Generation Research Institute, AD500 Andorra la Vella, Andorra; 5 Faculty of Sports Organization, Autonomous University of Nuevo Leon, San Nicolas de los Garza 66455, Nuevo Leon, Mexico; 6 Department of Physical Activity Sciences, Faculty of Education Sciences, Universidad Católica del Maule, Talca 3480112, Chile; exal.garcia@gmail.com; 7 School of Education, Faculty of Human Sciences, Universidad Bernardo O’Higgins, Santiago 8370993, Chile; 8 Universidad Autónoma del Paraguay, Asunción 001012, Paraguay; josemontenegro733@gmail.com; 9 Vicerrectoría de Investigación y Postgrado, Universidad de Los Lagos, Osorno 5311157, Chile; fiorella.quiroz@yahoo.com; 10 Department of Health Research, Icen Cognis, 38370 La Matanza de Acentejo, Spain; 11 Faculty of Physical Education, National Pedagogical University, Bogotá 480100, Colombia; babecerrap@pedagogica.edu.co; 12 School of Medicine, Universidad Espiritu Santo, Samborondón 092301, Ecuador; 13 Faculty of Health Sciences, Universidad Tecnológica Atlántico Mediterráneo—UTAMED, 29590 Malaga, Spain

**Keywords:** maximal fat oxidation, FAT_max_, body composition, allometric scaling, sex differences, indirect calorimetry

## Abstract

**Background**: Men display higher absolute fat oxidation rates during exercise than women, but whether fat-free mass (FFM) moderates the substrate–intensity relationship in a sex-specific manner remains untested. **Methods**: In this cross-sectional laboratory study, we evaluated 37 competitive athletes (20 women, 17 men; age 22 ± 5 years) using dual-energy X-ray absorptiometry (DXA) and indirect calorimetry during incremental treadmill exercise (538 min-by-min observations) to evaluate a sex × FFM × intensity interaction using linear mixed-effects models. **Results**: The primary sex × FFM × intensity interaction was non-significant (β=+0.014, 95 % CI −0.021 to +0.049; p=0.443). Absolute maximal fat oxidation (MFO) was higher in men (0.826±0.234 vs. $0.655\pm 0.175\;g\cdot \min^{-1}$; p=0.038), but it disappeared when normalised to FFM (15.45±4.19 vs. $15.74\pm 4.85\;\mathrm{mg}\cdot {\mathrm{kg}\;\mathrm{FFM}}^{-1}\cdot \min^{-1}$; p=0.847). Interestingly, a strong sex × adiposity (DXA body-fat percentage, %) interaction was identified (β=−0.440, q<0.001), with higher adiposity linked to higher fat oxidation in men (β=+0.339, p<0.001) but lower in women (β=−0.115, p=0.010). **Conclusions**: Adiposity was identified as the most significant sex-specific moderator, with evidence showing that it is associated with greater fat oxidation in men and lower fat oxidation in women. The male advantage in absolute fat oxidation reflected greater FFM, because MFO per kilogram of FFM did not differ between sexes; muscle oxidative capacity was not measured directly. However, the exploratory nature of the study requires confirmation in future research.

## 1. Introduction

Four lines of evidence frame the present study. The first concerns sex differences in substrate oxidation during exercise, which have been documented for decades. At matched relative intensities, women oxidise proportionally more fat and less carbohydrate than men, whereas men attain higher absolute rates [1,2,3]. The contrast is conventionally attributed to greater male skeletal muscle mass together with the actions of ovarian hormones, chiefly 17β-oestradiol and, often in the opposite direction, progesterone. These hormones act on adipose tissue lipolysis, on plasma fatty acid availability, and on intramyocellular triglyceride storage [1,4,5]. The contrast has been replicated across independent laboratories and cohorts [1,2,3,6,7]. Its quantitative and mechanistic bases, however, are not settled. It remains unclear how much of the difference reflects the oxidative capacity of each kilogram of active tissue and how much simply reflects the larger amount of metabolically active lean tissue in men. When maximal fat oxidation (MFO) is expressed relative to fat-free mass (FFM), the apparent male advantage attenuates and may even reverse in favour of women, as reported in 300 healthy adults [6] and in more than 1100 athletes [7], with skeletal muscle mitochondrial volume density and function differing less between sexes than whole-body rates would suggest [8].

The second line of evidence concerns body composition itself. The quantity of lean tissue, the degree and distribution of adiposity, and cardiorespiratory fitness are all associated with the capacity to oxidise fat, but their relative contributions remain contested [9,10]. Recent work has argued that the quality rather than the quantity of lean mass (encompassing factors such as mitochondrial density, oxidative enzyme activity, and capillary supply) is decisive, with cardiorespiratory fitness acting as a mediator [11], while other analyses identify fat mass and habitual physical activity as the dominant determinants of peak fat oxidation [12]. At the cellular level, women generally show a greater proportional area of type I fibres, whereas men have larger type II fibres [13,14]. Sex differences in fibre-specific metabolic responses to exercise have also been described [15]. For instance, in athletic populations the distribution of lean and fat mass differs markedly between sexes and disciplines [16], and therefore any sex contrast in substrate oxidation is embedded in a body composition contrast that must be handled explicitly.

The third line of evidence concerns intensity. Substrate selection changes non-linearly as exercise intensity rises, producing the characteristic inverted-U trajectory of fat oxidation. Two distinct indices describe this curve. MFO is the highest fat oxidation rate attained (g·min^−1^), whereas FAT_max_ is the exercise intensity, usually expressed as a percentage of peak oxygen uptake (%VO_2peak_), at which MFO occurs [17,18]. Both are used as markers of metabolic flexibility during exercise [17,19]. Both indices are sensitive to protocol design, nutritional state, and the stoichiometric equations applied, which generates substantial between-study variability [20], and normative values are therefore only interpretable within standardised procedures. Interest in these indices extends beyond descriptive physiology: MFO is related to endurance performance [21], and training prescribed at FAT_max_ has been proposed as a strategy to improve body composition and metabolic control, with documented effects in people with overweight and obesity [22], in middle-aged women [23], and in older women with type 2 diabetes [24].

The fourth line of evidence concerns training background, which modifies fat-oxidative capacity independently of sex: even 2 weeks of high-intensity interval training increase the capacity for fat oxidation in women [25], and endurance training alters mitochondrial and capillary density in a dose-dependent manner [26]. Samples drawn from heterogeneous disciplines therefore carry a source of variability that is not sex and that is compounded by hormonal status in female participants [27]. Identical relative aerobic power does not denote identical training status in women and men, because sex-specific classification frameworks place a given VO_2peak_ value in a higher performance tier in women [28]. Matching the sexes on relative VO_2peak_ may therefore compare better-conditioned women with less-conditioned men, a potential bias for any FFM-normalised contrast. Heterogeneous athletic samples are nonetheless relevant. Athletes from team, combat, and power sports rarely compete at intensities where fat is the main fuel, yet MFO and FAT_max_ are increasingly measured across sports [7,18]. They are used to set low-intensity conditioning and recovery zones and to support body mass management, which is critical in weight category sports such as weightlifting and combat sports [22]. Pooling disciplines also widens the range of FFM and adiposity within each sex, which a moderation analysis requires.

These four lines of evidence have been established largely in isolation, and almost always by reducing the entire substrate oxidation curve to a single metric, such as MFO or FAT_max_. Because of this, two consequences follow. The first concerns scaling. Oxygen uptake and MFO are conventionally normalised by simple division by body mass or FFM. However, this ratio standard assumes a strictly isometric (proportional) scale where the y-intercept is zero, an assumption that is rarely tested and frequently false in samples differing in body size [29,30]. Where it fails, a reported “sex difference”, or an equally reported absence of one, may be a property of the denominator rather than of the metabolism; adjusting maximal oxygen uptake for skeletal muscle mass has been shown to change the direction of sex comparisons in endurance athletes [31], and, in national team collegiate athletes, expressing aerobic capacity per kilogram of body mass created spurious associations between regional anthropometry and MFO [18]. The second consequence is inferential. Because sex differences are typically established by comparing sex-separated models, it remains untested whether body composition moderates the intensity–oxidation relationship differently in each sex, which is a formally distinct claim requiring an explicit three-way interaction term rather than a comparison of two sets of coefficients [32].

The unresolved question is therefore not whether men oxidise more fat, but whether the difference survives a defensible normalisation for the amount of metabolically active lean tissue and whether body composition shapes the substrate–intensity curve in a sex-specific way. The answer has direct practical consequences: if body composition moderates the curve sex-specifically, FAT_max_-guided prescription must be individualised by sex. If the sex difference is simply a lean mass difference, individualising by lean mass is sufficient and considerably simpler [33]. Repeated gas exchange measurements across an incremental test provide the resolution needed to address this issue, but this requires models that respect the nesting of observations within participants and that permit formal interaction testing, for which linear mixed-effects models are appropriate [34].

For all these reasons, we have developed this investigation to formulate the following hypotheses. H1 (primary). FFM moderates the relationship between fat oxidation rate (FAT_ox_) and exercise intensity differently in women and men (sex × FFM × intensity ≠ 0). This hypothesis rests on the sex differences in muscle fibre composition and intramyocellular lipid storage summarised above, which could make the contribution of each kilogram of lean tissue sex-specific [4,14]. H2 (secondary, stated a priori). Because the sample spans disciplines with different energetic demands, between-participant heterogeneity in substrate oxidation may itself differ by sex; dispersion is therefore characterised by sex before any sex difference in model performance is interpreted, rather than invoking heterogeneity post hoc to explain it. H3 (secondary). The sex difference in MFO is attenuated or abolished by appropriate normalisation to FFM, as expected if fat oxidation scales mainly with the amount of lean tissue [6,7,12]. Given the modest sample available from data from our laboratory, and the deliberate diversity of sporting backgrounds, the study is framed as exploratory.

## 2. Materials and Methods

### 2.1. Study Design

This was a cross-sectional laboratory study with a sex-comparative design, in which each participant completed a single standardised incremental treadmill test. Sex and body composition were not assigned by the investigators, so the design is observational in the epidemiological sense, even though the exercise protocol was controlled. Accordingly, the study is reported following the Strengthening the Reporting of Observational Studies in Epidemiology (STROBE) guidelines [35]. Gas exchange was sampled repeatedly within that single test; the resulting nesting of observations within participants, rather than repeated testing sessions, was handled with mixed-effects models (Section 2.5). Assessments were conducted between 1 June and 30 August 2025 at the Nutritional Assessment and Nutritional Care Laboratory (LECEN) of the Tonalá University Centre, University of Guadalajara (Tonalá, Jalisco, Mexico), under controlled conditions (20–25 °C, 50–60% relative humidity).

### 2.2. Participants and Eligibility

Thirty-seven competitive athletes (20 women and 17 men; age: 22 ± 5 years; range: 16–39) were evaluated. All competed at the national level and were assessed during the general preparation period, outside of the competitive and transition phases. To characterise the sample based on varying athletic demands and account for the heterogeneity of the disciplines, the sports were grouped according to the predominant energy system [36] into oxidative (triathlon, 10 km run; *n* = 10), glycolytic (middle-distance running, martial arts; *n* = 16), and phosphagen-dominant (weightlifting, sprinting, flag football; *n* = 11).

Eligibility criteria were derived from the question at hand, which factors modify substrate oxidation, and which must be controlled for a sex contrast to be interpretable, rather than from a generic comorbidity list. Inclusion criteria were (i) competitive athlete in active training; (ii) assessment during general preparation; and (iii) written informed consent, with parental consent and participant assent for those under 18 years. Exclusion criteria were (i) musculoskeletal injury preventing maximal running in the preceding 3 months; (ii) diagnosed metabolic or endocrine disease, or medication with documented effects on substrate metabolism (β-blockers, corticosteroids, metformin, thyroid agents); (iii) caffeine, carnitine, exogenous ketone, or ephedrine supplementation within 48 h; (iv) ketogenic diet or declared carbohydrate restriction or loading within 7 days; (v) pregnancy or lactation; and (vi) amenorrhoea or irregular cycles (<21 or >35 days) in the preceding 6 months. Elimination criteria were urine specific gravity (24 h USG) outside 1.018–1.024, resting respiratory exchange ratio (RER) > 0.95, and failure to reach RER ≥ 1.0.

Factors that could not be controlled are treated throughout as recognised sources of heterogeneity: exact menstrual cycle phase (only active menstruation was avoided), hormonal contraceptive use, quantified dietary intake, and quantified training volume and history. Sporting discipline is retained as a design feature and characterised quantitatively in Section 3.1.

### 2.3. Procedures

Participants attended a single morning session (06:00–08:00 h) following an overnight fast of 6 to 8 h. Prior to the visit, participants were instructed to maintain their habitual mixed diet (without acute carbohydrate loading or restriction) and abstain from alcohol and stimulant-containing foods and beverages (e.g., caffeine or energy drinks) for 48 h. Additionally, they were instructed to avoid physical exercise for 12 h (and strenuous exercise for 24 h) prior to testing. Hydration status was verified by digital refractometry (ATAGO^®^ PAL-10S; ATAGO CO., Ltd., Minato, Tokyo, Japan). Anthropometric measurements (body mass and stretched stature) followed the protocols of the International Society for the Advancement of Kinanthropometry and were performed by an International Society for the Advancement of Kinanthropometry (ISAK) Level 2 anthropometrist [37]; the technical error of measurement was below 1% for both variables.

Body composition was measured by dual-energy X-ray absorptiometry (DXA; Lunar iDXA, General Electric, Madison, WI, USA), calibrated daily, and performed in accordance with methodological recommendations for athletic populations [38], with standardised hand and limb positioning [39] and after verbal confirmation on arrival that participants had complied with the 12 h exercise restriction. This check was necessary because several athletes habitually trained early in the morning, and exercise-induced fluid redistribution alters DXA estimates [40]. An in-house repositioning precision study in 30 individuals, following the precision assessment approach of the International Society for Clinical Densitometry [41], yielded coefficients of variation of 0.73% for total fat mass and 0.86% for body fat percentage. The DXA was the sole body composition method used in the primary analysis. Multifrequency bioelectrical impedance (InBody 720, InBody Co., Cerritos, CA, USA) was also recorded and used only in a sensitivity analysis, consistent with evidence that impedance and DXA are not interchangeable for FFM in active young adults [42].

Pulmonary gas exchange was recorded breath by breath (PNOE, ENDO Medical, Palo Alto, CA, USA) after a familiarisation session. The incremental treadmill protocol (Horizon Fitness^®^ T202-06; Johnson Health Tech, Cottage Grove, WI, USA) began at 6 km·h^−1^ and increased by 2 km·h^−1^ every 3 min until voluntary exhaustion. Three-minute stages were chosen because they yield FAT_max_ values comparable to those obtained with longer stages [17,20]. No formal steady-state criterion, such as a plateau in VO_2_ or RER within each stage, was applied, and this is acknowledged as a limitation (Section 4.2). Fat and carbohydrate oxidation rates (g·min^−1^) were computed with the stoichiometric equations of Péronnet and Massicotte [43], based on the non-protein respiratory quotient framework [44]: FAT_ox_ = 1.695 × VO_2_ − 1.701 × VCO_2_ and CHO_ox_ = 4.344 × VCO_2_ − 3.061 × VO_2_, with gas volumes in L·min^−1^. Gas exchange data were averaged over consecutive 60 s intervals, yielding up to three observations per 3 min stage; these minute-by-minute observations were used for the substrate oxidation curves and the mixed-effects models. MFO and FAT_max_ were determined from the mean of the final 60 s of each stage, restricted to stages with RER ≤ 1.0, in line with recommendations for gas exchange estimation of substrate use [45]. FAT_max_ was defined operationally as the relative intensity (%VO_2peak_) of the discrete stage eliciting the highest observed fat oxidation rate, without curve fitting [20,46]. Observations above an RER of 1.0 were retained in the curves and models to preserve the full intensity range. Because the stoichiometric assumptions no longer hold at these intensities, fat oxidation estimates near maximal effort are interpreted as indicative only [44,45]. Heart rate was recorded continuously (Polar H10, Polar Electro, Kempele, Finland), and perceived exertion was rated with the Borg CR-10 scale [47].

### 2.4. Data Handling

Subject-level variables (anthropometry, DXA, bioelectrical impedance analysis [BIA], peak oxygen uptake) and minute-by-minute variables (relative intensity, oxidation rates, heart rate, perceived exertion) were held in complementary data structures and merged by participant identifier. The minute-by-minute structure was rectangular (22 1-minute observations × 37 participants = 814 cells); minutes not completed by a participant were stored as 0.0 for intensity and for both oxidation rates. These 276 structural zeros (33.9% of cells) were excluded, leaving 538 valid participant-minute observations (women 268, men 270; median 15 observations per participant, range 8–22, corresponding to test durations of 8 to 22 min).

### 2.5. Statistical Analysis

Analyses were performed in Python 3.12 with a global seed of 42. Rather than assume a ratio standard, the scaling exponent for oxygen uptake was estimated by log–log regression of absolute VO_2peak_ on FFM, with sex as a covariate and a sex × ln (FFM) term to test slope homogeneity, following the allometric modelling framework [29]. The VO_2peak_ is reported under five normalisations: absolute, ratio to body mass, ratio to FFM, allometric with the sample-derived exponent, and allometric with the theoretical exponent 0.67. The MFO is reported in absolute terms and normalised to FFM. Between-sex comparisons of participant characteristics, MFO, and FAT_max_ used Welch’s *t*-test, which does not assume equal variances, when data were approximately normal within each sex and used the Mann–Whitney *U* test otherwise. Sport category was compared with the χ^2^ test. Effect sizes are Hedges’ *g* for Welch tests and *r* for Mann–Whitney tests. Equality of dispersion was assessed with the Brown–Forsythe test. All tests were two-sided with α = 0.05.

The primary confirmatory analysis was a single linear mixed-effects model of FAT_ox_ with fixed effects for sex, FFM, intensity, intensity^2^, all two-way products, and the sex × FFM × intensity term, adjusted for age, with a by-participant random intercept. The quadratic intensity term was included because fat oxidation follows an inverted-U relation with exercise intensity [17,45]. A linear term alone cannot represent a rise to a peak followed by a decline, whereas the quadratic term captures this curvature. Continuous predictors were standardised, and the primary inference is the *p* value of the three-way term; no multiplicity correction was applied because this is a single pre-specified test. Pre-specified secondary analyses comprised sex differences in MFO (absolute and FFM-normalised) and in FAT_max_, and the ten two-way sex × {intensity, intensity^2^, FFM, body-fat %, BMI} interaction terms for FAT_ox_ and CHO_ox_, with Benjamini–Hochberg false discovery rate (FDR) control across the ten terms [48]. The resulting adjusted *p* values are reported as *q* values, and terms with *q* < 0.05 were considered significant. Exploratory analyses comprised dispersion by sex (Brown–Forsythe tests, coefficients of variation, multivariate spread), between- versus within-participant variance decomposition of FAT_ox_, sex-separated models, a single Gradient Boosting model with subject-wise GroupKFold cross-validation interpreted with SHAP values [49], and sensitivity analyses adding sport category as a covariate, substituting BIA-derived FFM and, for the between-sex comparison of VO_2peak_, fixing the allometric exponent at 0.67. The predictive model was used strictly to check whether a flexible learner recovers the same ordering of determinants as the primary model, in keeping with recommendations for embedding machine learning within a hypothetico-deductive framework [50]. Full coefficient tables for the sex-separated models, the sensitivity analyses, and the dispersion comparison are reported in Appendix A, and the specification of the predictive model in Appendix B.

This analytical hierarchy was defined after data collection, based on prior literature [9,10,12] and before the reported analyses were run. It is not a pre-registration and is not presented as one.

## 3. Results

### 3.1. Comparability of the Two Groups

Participant characteristics are presented in Table 1, grouped into three blocks according to their status in the design: variables for which the groups were matched, variables that are constitutive for the sex contrast, and variables that were not matched by design but were adjusted for statistically.

The allometric exponent of absolute VO_2peak_ on FFM was 0.579 (95% CI 0.225–0.933); adjusted for sex, it was 0.462. The slope did not differ between sexes (*p* = 0.365), justifying a common exponent, and the null hypothesis of isometry (b = 1) was rejected (*p* = 0.024), so the ratio standard is not appropriate in this sample. The confidence interval is wide and does not distinguish the sample estimate from the theoretical range of 0.67–0.75; both are therefore reported. Under either exponent, VO_2peak_ did not differ between sexes (*p* = 0.220 with 0.46 and *p* = 0.675 with 0.67; Table 1), so the interpretation of the sex comparison is the same whichever exponent is adopted. The apparent equivalence of VO_2peak_ between sexes proved to be a property of the denominator rather than of the athletes: in absolute terms, men were 21% higher (*p* = 0.009; *g* = −0.95), and the difference disappeared using every expression that accounted for lean mass (Table 1).

The distribution of disciplines did not differ between sexes (*p* = 0.778). Within each sex, sport category explained 18.1% of the variance in MFO in women (*p* = 0.349) and 8.6% in men (*p* = 0.531); neither reached significance. Sporting background was therefore balanced across the contrast and was not a detectable driver of fat-oxidative variability in this sample, although the study is underpowered to exclude a moderate effect.

### 3.2. Primary Analysis: Fat-Free Mass Does Not Moderate the Curve Sex-Specifically

The primary hypothesis was not supported. In the mixed model of FAT_ox_, the sex × FFM × intensity interaction was small and non-significant (*β* = +0.014, 95% CI −0.021 to +0.049; *p* = 0.443). The FFM × intensity interaction was present overall (*β* = −0.032; *p* = 0.024) and pointed in the same direction within each sex (women *β* = −0.018, 95% CI −0.037 to +0.001, *p* = 0.057; men *β* = −0.033, 95% CI −0.064 to − 0.002, *p* = 0.036), indicating a common rather than a sex-specific moderation: in both sexes, greater FFM was associated with a steeper decline in FAT_ox_ as intensity rose.

The result was robust to two sensitivity analyses. Adding sport category as a covariate left the term unchanged (*β* = +0.014; *p* = 0.441), as did substituting BIA-derived FFM (*β* = +0.005; *p* = 0.809); all sensitivity specifications are given in Appendix A, Table A2. The corresponding three-way term for carbohydrate oxidation was likewise non-significant (*β* = +0.029; *p* = 0.689).

### 3.3. Secondary Analyses

**Figure 1:**
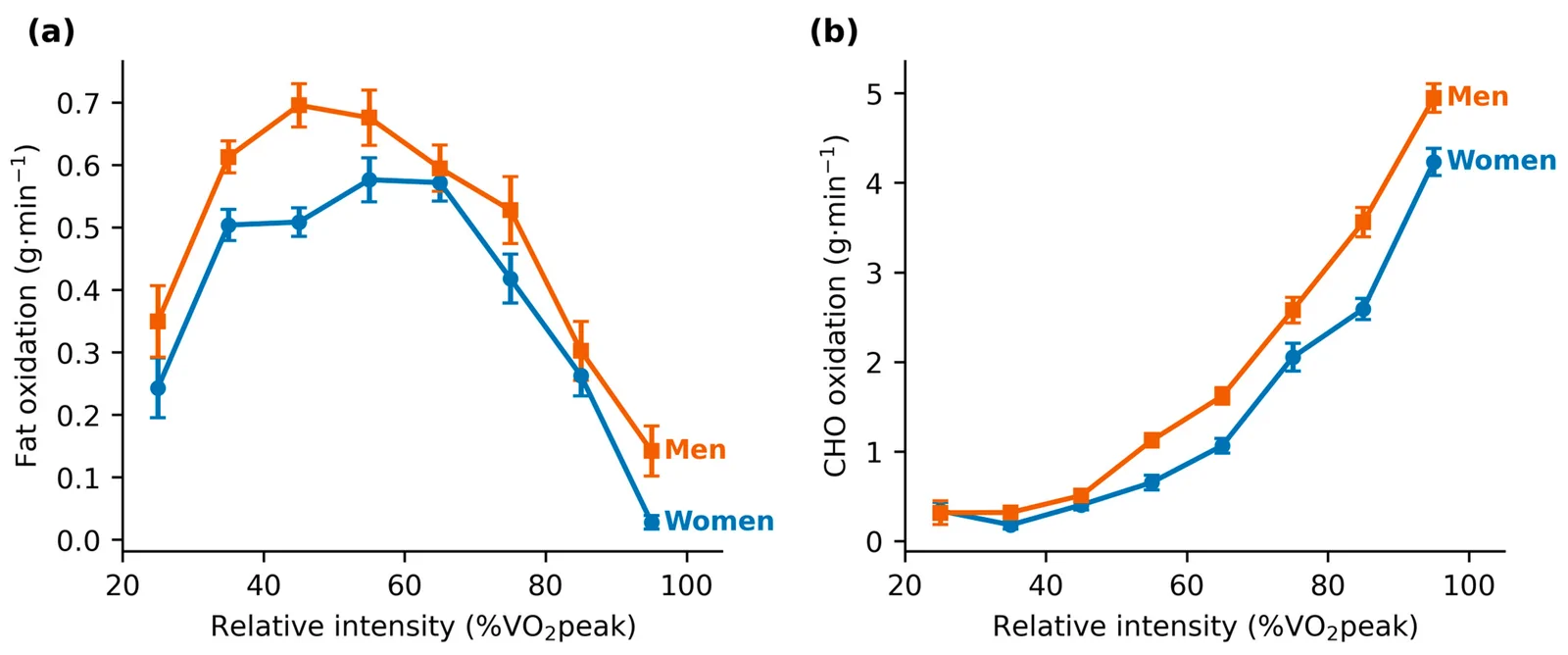
Substrate oxidation curves by sex across relative exercise intensity. (**a**) Fat oxidation; (**b**) carbohydrate oxidation. Points are means of 10%VO_2peak_ bins with standard errors; bins containing fewer than three observations are not shown. Series are labelled directly. *n* = 37 participants and 538 valid participant-minute observations. Colours follow the Okabe–Ito colourblind-safe palette. Bin means average participants whose individual peaks occur at different intensities and are therefore lower than the mean of individual MFO values reported in the text and in Figure 2. Observations above an RER of 1.0 are included, so fat oxidation estimates in the highest bins are indicative only.

**Figure 2:**
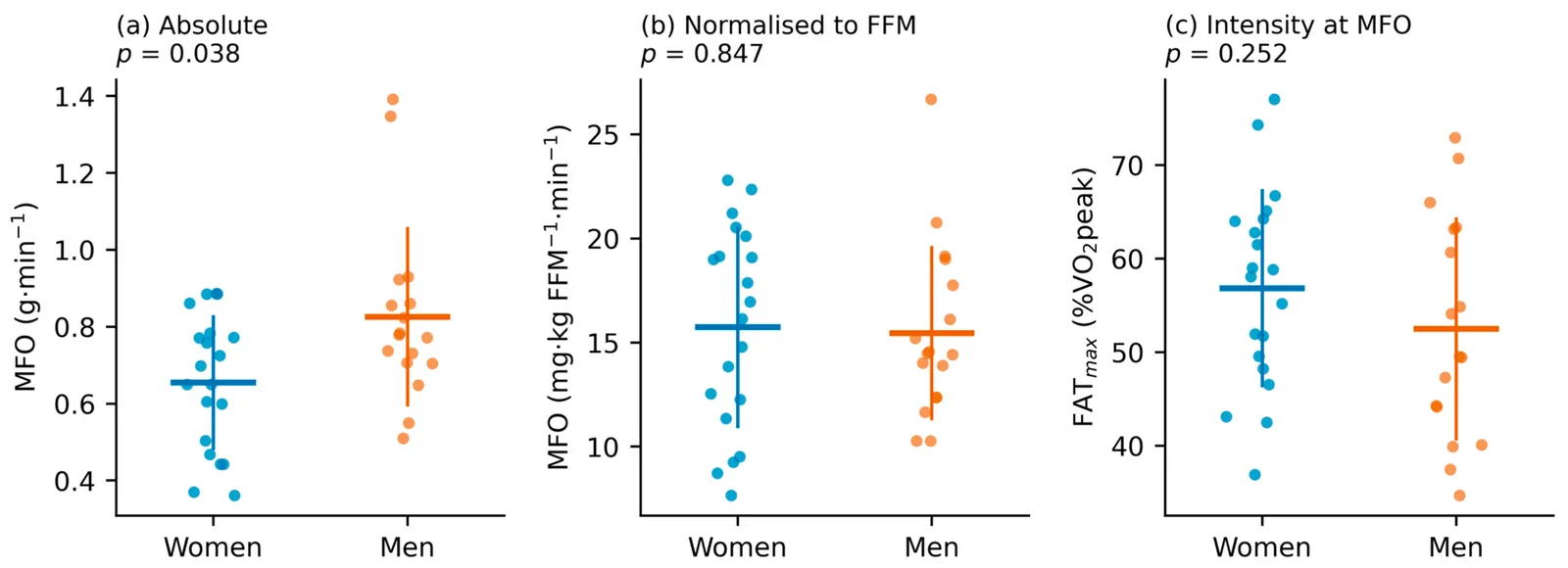
Maximal fat oxidation and its intensity by sex. (**a**) MFO in absolute terms; (**b**) MFO normalised to fat-free mass; (**c**) intensity at which MFO occurred. Points are individual participants, horizontally jittered; horizontal bars are means, and vertical bars are ± 1 standard deviation. *p* values are from Welch’s *t*-test, or from the Mann–Whitney *U* test where normality was not met. The contrast between panels (**a**) and (**b**) is the central finding of the study. Abbreviations: MFO, maximal fat oxidation; FFM, fat-free mass.

MFO was higher in men in absolute terms (0.826 ± 0.234 vs. 0.655 ± 0.175 g·min^−1^; *p* = 0.038; *g* = −0.84) but indistinguishable once normalised to FFM (15.45 ± 4.19 vs. 15.74 ± 4.85 mg·kg FFM^−1^·min^−1^; *p* = 0.847; *g* = +0.06). FAT_max_ did not differ (52.5 ± 11.9 vs. 56.8 ± 10.6%VO_2peak_; *p* = 0.252). Hypothesis H3 is therefore supported. Both sexes described the classic inverted-U FAT_ox_ trajectory and a monotonic rise in carbohydrate oxidation, with men higher in absolute terms across the intensity range (Figure 1); the contrast between absolute and normalised MFO is shown in Figure 2. The group curves in Figure 1a peak below the mean individual MFO (approximately 0.70 vs. 0.83 g·min^−1^ in men and 0.58 vs. 0.66 g·min^−1^ in women). This is expected, because each bin averages participants whose individual maxima occur at different intensities. Individual FAT_max_ values spanned more than 35 percentage points of VO_2peak_ within each sex (Figure 2c).

**Figure 3:**
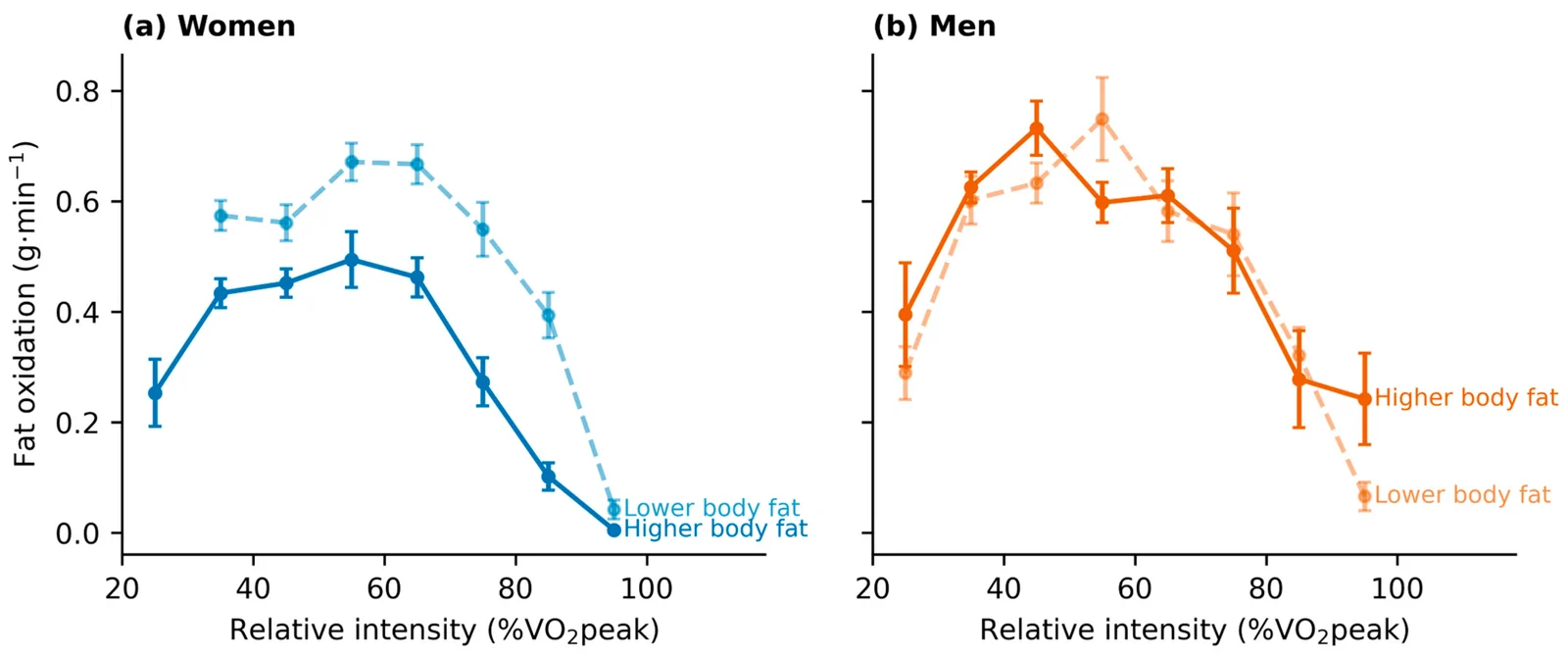
Sex-specific moderation of fat oxidation by adiposity. Fat oxidation curves within (**a**) women and (**b**) men, split at the within-sex median of DXA body fat percentage. Groups comprised 10 lower and 10 higher body fat women and 9 lower and 8 higher body fat men. Points are means of 10%VO_2peak_ bins with standard errors. Higher adiposity was associated with lower fat oxidation in women and with higher fat oxidation in men (sex × body fat%: *q* < 0.001). Abbreviations: DXA, dual-energy X-ray absorptiometry.

As shown in Table 2, the sex × body fat percentage interaction on FAT_ox_ was the largest term in the secondary model (*β* = −0.440; *q* < 0.001), and its direction was opposite in the two sexes: within men, higher body fat percentage was associated with higher FAT_ox_ (*β* = +0.339, 95% CI +0.159 to +0.519; *p* < 0.001), whereas within women it was associated with lower FAT_ox_ (*β* = −0.115, 95% CI −0.202 to −0.028; *p* = 0.010). Figure 3 displays the corresponding curves. Carbohydrate oxidation rose more steeply with intensity in men (sex × intensity *β* = −0.296; *q* < 0.001).

**Table 2:** Sex × variable interaction terms for fat and carbohydrate oxidation (538 observations, 37 participants), with Benjamini–Hochberg *q* values.

Outcome	Interaction	*β*	95% CI	*p*-Value	*q*
FAT_ox_	Sex × body fat%	−0.440	−0.629, −0.250	<0.001	<0.001
FAT_ox_	Sex × BMI	+0.305	+0.117, +0.493	0.0014	0.005
FAT_ox_	Sex × FFM	−0.280	−0.464, −0.096	0.0029	0.007
FAT_ox_	Sex × intensity^2^	−0.010	−0.031, +0.012	0.395	0.493
FAT_ox_	Sex × intensity	+0.008	−0.020, +0.035	0.570	0.634
CHO_ox_	Sex × intensity	−0.296	−0.406, −0.185	<0.001	<0.001
CHO_ox_	Sex × BMI	−0.781	−1.411, −0.152	0.015	0.030
CHO_ox_	Sex × body fat%	+0.701	+0.066, +1.336	0.030	0.051
CHO_ox_	Sex × FFM	+0.442	−0.176, +1.060	0.161	0.230
CHO_ox_	Sex × intensity^2^	−0.005	−0.092, +0.083	0.918	0.918

Note: Sex was coded 1 = woman and 0 = man. Continuous predictors were standardised; all models included a by-participant random intercept and were adjusted for age. *q* values indicate terms surviving false discovery rate control at 5%. Abbreviations: BMI, body mass index; CHO_ox_, carbohydrate oxidation; CI, confidence interval; FAT_ox_, fat oxidation; FFM, fat-free mass.

### 3.4. Exploratory Analyses

Women were not more homogeneous than men. Brown–Forsythe tests were non-significant for body mass, stature, BMI, FFM, body fat percentage, VO_2peak_, and MFO (all *p* ≥ 0.12); the only significant difference was in age, where women were the more dispersed group (*p* = 0.013). Multivariate spread, computed as the mean Euclidean distance to the own-sex centroid in standardised units across mass, stature, FFM, body fat percentage, and VO_2peak_, was 1.62 in women and 1.88 in men. Variance decomposition of FAT_ox_ itself, however, showed a clear asymmetry: between-participant variance was 3.7 times larger in men (0.0445 vs. 0.0120), giving intraclass correlation coefficients (ICCs) of 0.38 in men and 0.17 in women. The full dispersion comparison is reported in Appendix A, Table A3, and the sex-separated model coefficients in Appendix A, Table A1.

**Figure A1:**
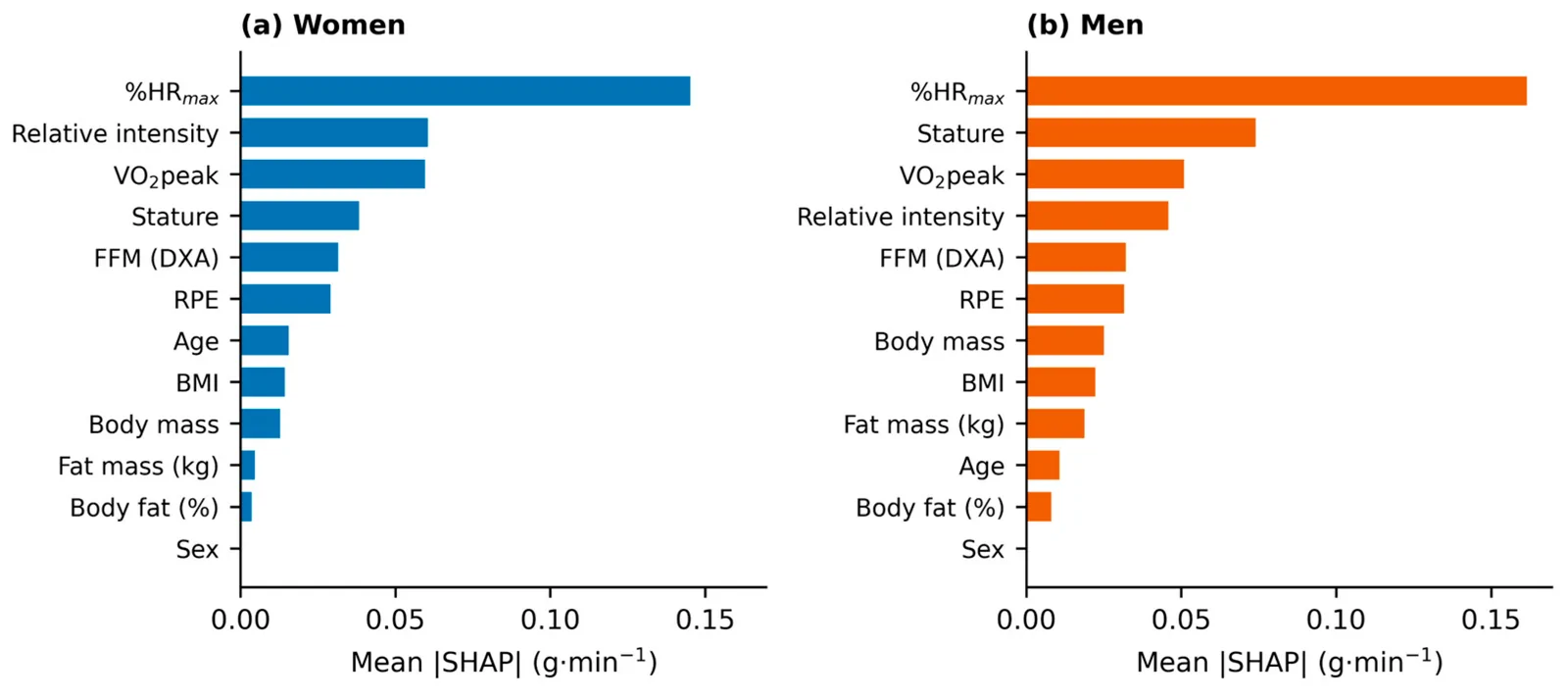
Mean absolute SHAP values by sex for the exploratory Gradient Boosting model of fat oxidation. (**a**) Women; (**b**) men. Bars represent the mean absolute contribution of each predictor to the model output, in g·min^−1^. %HRmax ranked first in both sexes; subsequent ranks differed (relative intensity second in women, stature second in men), but the same five predictors occupied the first five positions in both sexes. Abbreviations: %HRmax, percentage of maximal heart rate; BMI, body mass index; DXA, dual-energy X-ray absorptiometry; FFM, fat-free mass; RPE, rating of perceived exertion; SHAP, SHapley Additive exPlanations; VO_2peak_, peak oxygen uptake.

A single Gradient Boosting model with subject-wise GroupKFold cross-validation explained just under half of the variance in FAT_ox_ overall (*R*^2^ = 0.465; RMSE = 0.223 g·min^−1^), with better performance in women (*R*^2^ = 0.664; RMSE = 0.152) than in men (*R*^2^ = 0.322; RMSE = 0.275). This ordering is consistent with the variance decomposition above, since subject-wise cross-validation must extrapolate the between-participant component, which is larger in men. A by-participant cluster bootstrap (2000 resamples) gave 95% confidence intervals of 0.502–0.768 in women and 0.035–0.554 in men and +0.08 to +0.65 for the difference, an interval so wide that the difference is reported as hypothesis-generating only. SHAP attribution placed relative heart rate first in both sexes by a wide margin; in women it was followed by intensity, VO_2peak_, stature and FFM, whereas in men stature ranked second, followed by VO_2peak_, intensity and FFM. The same five predictors therefore occupied the first five positions in both sexes, and no body-composition variable rose to prominence in one sex only, which is consistent with the null primary result (Appendix B, Figure A1).

## 4. Discussion

Contrary to our primary hypothesis (H1), fat-free mass did not moderate the fat oxidation–intensity relationship differently in women and men: the three-way interaction was small and non-significant, unchanged by adjustment for sporting discipline or by substituting an alternative measurement of lean mass, and, within each sex, the FFM × intensity term pointed in the same direction. Whatever role lean mass plays in shaping the substrate curve during incremental exercise, the present data provide no evidence that it is sex-specific. This null result is reported as our principal finding rather than displaced by significant secondary terms, in keeping with the requirement that interaction claims be tested explicitly rather than inferred from side-by-side models [32,34]. Consequently, our findings resolve three key points relative to our initial hypotheses: first, FFM moderation of the fat oxidation–intensity curve is not sex-dependent (H1); second, marked variance in substrate oxidation was not driven by greater female homogeneity, though men displayed broader between-participant variability in fat oxidation (H2); and third, the apparent male advantage in peak fat oxidation was fully abolished when normalised to FFM (H3). The overarching observation that greater lean mass accelerates the drop in fat oxidation across both sexes aligns with the classical description of the intensity-dependent crossover in substrate use [17,19] and reinforces the view that this metabolic shift is governed by the demand placed on the active muscle mass rather than by sex per se [2,4].

The observed sex difference in fat oxidation reduces fundamentally to lean mass. Absolute MFO was 0.17 g·min^−1^ higher in men. However, this difference vanished entirely when normalised to FFM. Similarly, FAT_max_ showed no sex difference. The same pattern applied to aerobic capacity. Absolute VO_2peak_ was 21% higher in men but reached equivalence under all scaled expressions. Together, these results indicate that the male advantage reflects the larger amount of metabolically active lean tissue in men rather than a greater fat-oxidative capacity per kilogram of that tissue. This finding aligns with several lines of literature. First, the male advantage attenuates when MFO is scaled to lean mass [6,7]. Second, FFM is a primary determinant of MFO in large cohorts [12,51]. Third, sex differences in skeletal muscle mitochondrial density and function are far smaller than whole-body rates suggest [8,26]. Furthermore, women exhibit higher relative fat reliance as a fraction of energy expenditure, even when absolute rates favour men [1,3]. Finally, MFO scales consistently with lean tissue across training backgrounds [9,10]. These data address the debate on lean mass quantity versus quality [11]: once quantity is accounted for, no residual sex difference remains to be explained by tissue quality. This interpretation requires one qualification. Women generally show a greater proportional area of type I fibres and store more intramyocellular triglyceride [4,13,14], which could raise fat-oxidative capacity per kilogram of muscle. Such an advantage would make FFM-normalised MFO favour women, as reported in some large cohorts [6,7], yet no such advantage appeared here. Two considerations apply. First, DXA-derived FFM includes organs, skin, connective tissue, and bone mineral, as well as skeletal muscle, so it is an imperfect proxy for the active muscle mass. Second, muscle fibre composition, capillarisation, and oxidative enzymes were not measured [8,15], so any difference in tissue quality could not be quantified. Muscle biopsies or imaging would be needed to test these mechanisms directly.

The methodological corollary deserves emphasis, because it is the reason the finding is visible at all. Ratio standards are valid only under isometric conditions, and here the exponent relating VO_2peak_ to FFM was significantly below unity, as allometric analyses of oxygen uptake have repeatedly shown for body mass [29,30]. Reporting similar VO_2peak_ between sexes on the basis of mL·kg^−1^·min^−1^, as is conventional, would have described a property of the denominator as though it were a property of the athletes; adjusting for skeletal muscle mass has previously been shown to reverse the direction of such comparisons in endurance athletes [31]. The same caution applies to MFO, which is why both expressions are reported here, and it applies more generally to the interpretation of sex comparisons in body composition-dependent variables in sport [16,33,52].

The strongest sex-specific moderator identified was adiposity, and it acted in opposite directions. Higher body fat percentage was associated with higher fat oxidation in men and lower fat oxidation in women. The positive association in men aligns with observations that exercise fat oxidation increases with body fatness in men with obesity, contrary to the metabolic inflexibility paradigm [53], and with the broader dependence of fat-oxidative rates on substrate availability [54]. Several mechanisms are compatible with the divergence between sexes. Men and women differ in regional fat distribution and in the lipolytic responsiveness of these adipose depots [55,56]. These differences extend to adrenergic signalling and circulating fatty acid kinetics [5,57,58]. Women also store more intramyocellular triglycerides and express higher levels of perilipin proteins [59,60]. Consequently, female fat oxidation may rely more on intramuscular sources and less on circulating fatty acids [59,60]. Beyond substrate availability, sex differences in the active musculature, such as fibre-type proportion, capillary density, and mitochondrial characteristics, may further shape these metabolic responses [8,61]. Resting skeletal-muscle lipase and carnitine palmitoyltransferase content correlate with peak fat oxidation in both sexes, though with different weightings [61]. Because neither active muscle kinetics nor tissue-level enzymes were measured here, these mechanistic interpretations remain hypothetical. Regional fat distribution may add a further, sex-specific layer. In men, a higher body fat percentage within this athletic range is likely to reflect larger abdominal subcutaneous and visceral depots, which respond readily to catecholamine-stimulated lipolysis and could raise plasma fatty acid supply during exercise [55,56]. In women, gluteofemoral subcutaneous fat predominates. This depot shows greater α_2_-adrenergic antilipolytic responsiveness and may release fatty acids less readily during exercise [55,58]. Regional fat mass and lipolytic flux were not analysed here, so this explanation remains speculative. Finally, the adiposity term did not yield a significant three-way interaction with intensity. Thus, any potential effect reflects a shift in the overall level of the fat oxidation curve rather than a change in its shape.

Heterogeneity and predictability were addressed by design rather than post hoc. The characterisation gave a two-part answer. Women were not more homogeneous in body composition or aerobic fitness; if anything, they were more variable in age, so an explanation of differential model performance in terms of a more uniform female group is not supported. Men, however, showed almost four times the between-participant variance in fat oxidation itself. Because subject-wise cross-validation requires extrapolation to an unseen participant, precisely this component determines out-of-sample error, which is consistent with the observed ordering of *R*^2^. The magnitude of that between-participant component also constrains how precisely MFO can be estimated in any single individual [20,62]. Such between-individual variability in fat oxidation is well documented even under standardised conditions [10,62], and it is the reason that predictive performance in this setting should be interpreted as a property of the sample structure rather than of the biology [50]. The bootstrap interval around the difference was very wide, and no robust sex difference in predictability is claimed. The same dispersion bears on the null primary result. Individual FAT_max_ values spanned more than 35 percentage points of VO_2peak_ within each sex, and differences in cardiorespiratory fitness and muscle conditioning may have added variance that could mask a modest sex-specific moderation by FFM. In a recent study of national team collegiate athletes, absolute VO_2peak_ explained about 60% of the variance in MFO, whereas FFM and regional anthropometry added no independent information [18]. Gross FFM, fat mass, and VO_2peak_ may therefore act as correlates or confounders of individual FAT_max_ rather than as its primary determinants. Group-based FAT_max_ targets derived from sex or body composition would then be imprecise, and individual testing remains necessary for prescription [20,63].

Several unmeasured factors deserve explicit mention. Ovarian hormones influence lipid metabolism during exercise, where oestrogen generally promotes lipolysis and fatty acid availability, while progesterone can exert antagonistic effects [1,27,64]. However, the magnitude of these substrate shifts across menstrual cycle phases during acute exercise remains small and variable in the literature [1,27]. Because menstrual cycle phase and oral contraceptive use were not standardised or recorded here, residual hormonal variance could not be modeled, and part of the between-woman variability observed may reflect hormonal fluctuation rather than body composition. Even so, large cohort studies indicate that menstrual phase accounts for negligible variance in MFO compared to lean mass [12,51]. The magnitude of that contribution remains contested. A previous work reports measurable shifts in substrate oxidation across natural cycle and oral-contraceptive phases [65], whereas recent evidence indicates that peak fat oxidation during submaximal exercise remains largely consistent across cycle phases and combined oral contraceptive use [66]. Current position statements nonetheless recommend that cycle phase be documented in female athlete research [67,68], and the same applies to the reporting of body composition methodology, since method choice alone can shift lean mass estimates by several kilograms in athletic samples [38,52]. Training background was captured only as a coarse categorisation by predominant energy system, without training volume, years of practice, or threshold characterisation, even though relatively short training exposures are sufficient to alter fat-oxidative capacity [25,26].

Finally, the estimation of substrate oxidation from gas exchange carries assumptions that are well characterised but not eliminable. Stoichiometric equations assume negligible protein oxidation and a stable bicarbonate pool, conditions that are progressively violated as intensity rises [44,45]; short stage durations can underestimate carbohydrate oxidation [69]; tracer-based comparisons show that gas exchange captures whole-body but not source-specific fluxes [70]; and metabolic carts themselves differ measurably in the accuracy of derived substrate estimates [71]. Restricting the determination of MFO and FAT_max_ to stages with an RER of 1.0 or less, as done here, limits but does not remove these concerns. The incremental protocol also bears on exercise prescription. Three-minute stages may not allow full adjustment of gas exchange kinetics and bicarbonate stores, and FAT_max_ derived from incremental tests does not always reproduce the fat oxidation observed during prolonged constant-load exercise at that intensity [63]. Incremental testing is appropriate for describing the whole substrate curve and comparing groups, which was the purpose here. Training zones derived from it should nonetheless be verified with prolonged steady-state bouts at the prescribed intensities [20,63].

### 4.1. Practical Implications

These are cross-sectional, observational data, and what follows is not a demonstrated effect. If the sex difference in fat oxidation is essentially a lean mass difference, then individualising FAT_max_-guided prescription by lean mass would capture most of what individualising by sex would achieve, which is operationally simpler and avoids applying group-average sex corrections to individuals. The possible exception concerns adiposity, whose moderation of the curve did differ by sex; whether that translates into different optimal training intensities is untested. Given the documented benefits of FAT_max_-based training on body composition and cardiometabolic risk [22,23,24], this is a question worth resolving prospectively.

### 4.2. Limitations

The sample is small (20 women, 17 men), and the study is explicitly exploratory, so the null primary result should be read as an absence of evidence within an underpowered design rather than as evidence of absence; detecting interaction terms requires substantially larger samples than detecting main effects [72]. The analytical hierarchy was defined after data collection, based on prior literature but without pre-registration. The cross-sectional design precludes causal inference. Menstrual cycle phase, hormonal contraception, dietary intake, and training load were not recorded. The allometric exponent was estimated with wide uncertainty, although conclusions were unchanged when the theoretical exponent was imposed. Predictive models were assessed only by internal, subject-wise cross-validation and await external confirmation. The age range spans adolescence to early middle age, and maturation-related differences in substrate oxidation cannot be excluded in the youngest participants. Generalisation beyond trained individuals requires separate study. Participants were matched for VO_2peak_ relative to body mass (about 51 mL·kg^−1^·min^−1^ in both sexes), but this value corresponds to a higher performance level in women than in men using sex-specific classification frameworks [28]. The contrast may therefore compare relatively better-conditioned women with moderately trained men. Because training raises fat-oxidative capacity [25,26], this conditioning bias would tend to favour women in normalised comparisons. The primary model was not adjusted for VO_2peak_, which is itself strongly related to FFM, so residual confounding by cardiorespiratory fitness cannot be excluded. The 6–8 h overnight fast was shorter than the 10–12 h period commonly used in metabolic studies, and diet was neither standardised nor recorded during the preceding 24 h. Differences in muscle and liver glycogen status may therefore have added between-participant variance in substrate oxidation [20]. No formal steady-state criterion was applied within the 3 min stages. Finally, ventilatory and lactate thresholds were not determined, although they would have described aerobic conditioning more fully and allowed FAT_max_ to be located relative to individual metabolic transitions [73].

### 4.3. Future Directions

When considering these preliminary findings, several targeted directions emerge for future research. Adequately powered studies with participants matched for training volume, and with menstrual cycle phase controlled or recorded, are required to confirm whether the sex-specific adiposity moderation identified here is robust. Furthermore, stable isotope tracers and muscle imaging would allow our lean mass interpretation to be tested directly by quantifying the relative contributions of plasma and intramuscular fatty-acid sources [59,70]. Finally, whenever sex comparisons of oxidative variables are reported, underlying scaling assumptions must be tested explicitly rather than assumed [29]. Future protocols should also include ventilatory or lactate thresholds, a 10–12 h fast with dietary standardisation, and verification of FAT_max_ during prolonged steady-state exercise. DXA-derived android and gynoid fat mass could be used to test whether regional adiposity explains the sex-specific association between body fat and fat oxidation.

## 5. Conclusions

In athletes drawn from diverse sporting disciplines, fat-free mass did not moderate the fat oxidation–intensity relationship in a sex-specific way. The male advantage in maximal fat oxidation was fully accounted for by fat-free mass, and the intensity at which fat oxidation peaked did not differ between sexes. The strongest sex-specific moderator identified was adiposity, which was associated with higher fat oxidation in men and lower fat oxidation in women; this finding is exploratory and requires confirmation. For practice, these data suggest that individualising FAT_max_-guided prescription by lean mass may capture most of what individualising by sex would achieve, except for adjustments related to adiposity.

## Figures and Tables

**Table 1:** Participant characteristics by sex (20 women, 17 men), organised by comparability status.

Variable	Women (*n* = 20)	Men (*n* = 17)	Test	*p*-Value	Effect Size
(a) Matched—no meaningful difference					
BMI (kg·m^−2^)	21.2 ± 1.8	22.2 ± 3.1	Welch *t*	0.218	*g* = −0.42
Fat mass, DXA (kg)	13.5 ± 3.1	12.4 ± 4.5	Welch *t*	0.401	*g* = +0.28
VO_2peak_/body mass (mL·kg^−1^·min^−1^)	51.2 ± 9.2	51.3 ± 11.1	Welch *t*	0.960	*g* = −0.02
VO_2peak_/FFM (mL·kg FFM^−1^·min^−1^)	68.4 ± 13.5	64.8 ± 13.1	Welch *t*	0.411	*g* = +0.27
VO_2peak_/FFM^−0.46 ^(mL·kg FFM^−0.46^·min^−1^)	510 ± 85	551 ± 109	Welch *t*	0.220	*g* = −0.41
VO_2peak_/FFM^−0.67 ^(mL·kg FFM^−0.67^·min^−1^)	234 ± 41	240 ± 47	Welch *t*	0.675	*g* = −0.14
FAT_max_ (%VO_2peak_)	56.8 ± 10.6	52.5 ± 11.9	Welch *t*	0.252	*g* = +0.39
Sport category (oxidative/glycolytic/phosphagen)	6/9/5	4/7/6	*χ* ^2^	0.778	—
(b) Not matched—constitutive for the sex contrast					
Body mass (kg)	56.5 ± 6.3	68.2 ± 9.8	Mann–Whitney	<0.001	*r* = 0.69
Stretch stature (cm)	163.3 ± 6.7	175.1 ± 7.0	Welch *t*	<0.001	*g* = −1.69
FFM, DXA (kg)	42.7 ± 7.1	53.8 ± 7.1	Welch *t*	<0.001	*g* = −1.53
Body fat, DXA (%)	24.3 ± 4.9	17.4 ± 5.8	Welch *t*	<0.001	*g* = +1.27
VO_2peak_, absolute (L·min^−1^)	2.9 ± 0.5	3.5 ± 0.7	Welch *t*	0.009	*g* = −0.95
(c) Not matched by design—adjusted for statistically					
Age (y)	23.3 ± 6.2	20.1 ± 2.7	Mann–Whitney	0.145	*r* = 0.28

Note: values are mean ± standard deviation. Tests were Welch’s *t*-test or the Mann–Whitney *U* test according to within-sex normality; effect sizes are Hedges’ *g* and *r*, respectively. The pooled mean age (21.8 years, reported as 22 ± 5 years in the text) equals the participant-weighted mean of the sex-specific values. Although central tendency in age did not differ, dispersion did (Brown–Forsythe *p* = 0.013), with women being the more variable group. Menstrual cycle phase, hormonal contraceptive use, quantified dietary intake, and quantified training load were not recorded. Abbreviations: BMI, body mass index; DXA, dual-energy X-ray absorptiometry; FFM, fat-free mass; VO_2peak_, peak oxygen uptake.

## Data Availability

De-identified data supporting the conclusions of this article will be made available by the authors on reasonable request.

## Abbreviations

The following abbreviations are used in this manuscript:

BIA	Bioelectrical impedance analysis
BMI	Body mass index
CHO	Carbohydrate
CHO_ox_	Carbohydrate oxidation rate
CI	Confidence interval
CV	Coefficient of variation
DXA	Dual-energy X-ray absorptiometry
FAT_max_	Relative exercise intensity eliciting maximal fat oxidation
FAT_ox_	Fat oxidation rate
FDR	False discovery rate
FFM	Fat-free mass
ICCs	Intraclass correlation coefficients
ISAK	International Society for the Advancement of Kinanthropometry
LECEN	Nutritional Assessment and Nutritional Care Laboratory
MFO	Maximal fat oxidation
RER	Respiratory exchange ratio
RMSE	Root mean square error
RPE	Rating of perceived exertion
SD	Standard deviation
SHAP	SHapley Additive exPlanations
STROBE	Strengthening the Reporting of Observational Studies in Epidemiology
USG	Urine specific gravity
VCO_2_	Carbon dioxide production
VO_2_	Oxygen uptake
VO_2peak_	Peak oxygen uptake

## Appendix A. Supplementary Statistical Analyses

### Appendix A.1. Sex-Separated Mixed-Effects Models

The primary analysis reported in Section 3.2 tests a single three-way interaction and is the only confirmatory contrast in the study. Because sex-separated models are widely reported in this literature, and because their side-by-side comparison is precisely the inference that the primary model was designed to replace, the corresponding within-sex coefficients are provided here in full so that readers can verify that no coefficient pattern was suppressed. Table A1 shows that the fat-free mass × intensity term carries the same sign in both sexes, which is the basis for the conclusion that the moderation is common rather than sex-specific.

**Table A1:** Sex-separated linear mixed-effects models for fat and carbohydrate oxidation.

Outcome	Term	Women, *β* (95% CI)	Women, *p*	Men, *β* (95% CI)	Men, *p*
FAT_ox_	Intensity	−0.223 (−0.244, −0.201)	<0.001	−0.200 (−0.227, −0.172)	<0.001
FAT_ox_	Intensity^2^	−0.161 (−0.173, −0.148)	<0.001	−0.152 (−0.170, −0.134)	<0.001
FAT_ox_	FFM	−0.091 (−0.198, +0.016)	0.097	+0.229 (+0.072, +0.385)	0.004
FAT_ox_	Body fat %	−0.115 (−0.202, −0.028)	0.010	+0.339 (+0.159, +0.519)	<0.001
FAT_ox_	BMI	+0.060 (−0.044, +0.163)	0.258	−0.260 (−0.420, −0.100)	0.002
FAT_ox_	Age	−0.028 (−0.077, +0.021)	0.264	−0.142 (−0.300, +0.016)	0.079
FAT_ox_	FFM × intensity	−0.018 (−0.037, +0.001)	0.057	−0.033 (−0.064, −0.002)	0.036
CHO_ox_	Intensity	+1.697 (+1.605, +1.789)	<0.001	+1.808 (+1.704, +1.912)	<0.001
CHO_ox_	Intensity^2^	+0.512 (+0.458, +0.566)	<0.001	+0.519 (+0.451, +0.587)	<0.001
CHO_ox_	FFM	+0.561 (+0.155, +0.967)	0.007	+0.137 (−0.359, +0.633)	0.588
CHO_ox_	Body fat %	+0.192 (−0.138, +0.521)	0.254	−0.500 (−1.069, +0.070)	0.086
CHO_ox_	BMI	−0.379 (−0.770, +0.012)	0.058	+0.379 (−0.128, +0.886)	0.143
CHO_ox_	Age	+0.029 (−0.157, +0.214)	0.763	−0.131 (−0.632, +0.370)	0.608
CHO_ox_	FFM × intensity	+0.163 (+0.082, +0.245)	<0.001	+0.136 (+0.020, +0.253)	0.022

Predictors were standardised (mean 0, SD 1); all models included a by-participant random intercept. Sample sizes are 268 participant-minute observations in women and 270 in men. Abbreviations: BMI, body mass index; CHO_ox_, carbohydrate oxidation; CI, confidence interval; FAT_ox_, fat oxidation; FFM, fat-free mass.

### Appendix A.2. Sensitivity Analyses of the Primary Interaction

Sensitivity analyses were pre-specified to establish that the null primary result is not an artefact of a single modelling decision (Table A2). None altered the conclusion. The sex × FFM × intensity term remained small and non-significant with adjustment for sporting discipline and with substitution of the body composition method, and the equivalent term for carbohydrate oxidation was also non-significant. Because the primary model does not include peak oxygen uptake, the allometric exponent does not enter it; the exponent affects only the between-sex comparison of VO_2peak_, which was non-significant with both exponents (Table 1).

**Table A2:** Sensitivity analyses for the primary sex × FFM × intensity interaction on fat oxidation.

Model Specification	*β*	95% CI	*p*
Primary model (Section 3.2)	+0.014	−0.021, +0.049	0.443
+Sport category as covariate	+0.014	−0.021, +0.049	0.441
FFM from BIA instead of DXA	+0.005	−0.035, +0.045	0.809
Same term for carbohydrate oxidation	+0.029	−0.112, +0.169	0.689

All models were adjusted for age and included a by-participant random intercept. Abbreviations: BIA, Bioelectrical impedance analysis; CI, confidence interval; DXA, dual-energy X-ray absorptiometry; FFM, fat-free mass.

### Appendix A.3. Dispersion by Sex

Hypothesis H2 required that between-participant heterogeneity be characterised before any sex difference in model performance was interpreted. Table A3 reports the full dispersion comparison summarised in Section 3.4. Equality of dispersion was rejected only for age, and in the direction opposite to the one that would explain the observed ordering of model performance.

**Table A3:** Coefficients of variation and equality-of-dispersion tests by sex.

Variable	CV Women (%)	CV Men (%)	Brown–Forsythe *p*
Age	26.5	13.4	0.013
Body mass	11.2	14.4	0.167
Stretch stature	4.1	4.0	0.675
BMI	8.6	13.8	0.125
FFM (DXA)	16.6	13.1	0.961
Body fat (DXA)	20.3	33.7	0.381
VO_2peak_	18.0	21.7	0.569
MFO	26.8	28.3	0.165

Multivariate spread, computed as the mean Euclidean distance to the own-sex centroid in standardised units across body mass, stature, FFM, body fat percentage, and VO_2peak_, was 1.62 in women and 1.88 in men. Abbreviations: BMI, body mass index; CV, coefficient of variation; DXA, dual-energy X-ray absorptiometry; FFM, fat-free mass; MFO, maximal fat oxidation; VO_2peak_, peak oxygen uptake.

## Appendix B. Exploratory Predictive Model

The Gradient Boosting model reported in Section 3.4 was fitted with default hyperparameters and a fixed random seed of 42, using subject-wise GroupKFold cross-validation with five folds so that no participant contributed observations to both the training and the test partition of a given fold. Predictors were relative intensity, rating of perceived exertion, percentage of maximal heart rate, sex, fat-free mass, body fat percentage, absolute fat mass, body mass index, age, peak oxygen uptake, body mass, and stature. The model was interpreted with SHapley Additive exPlanations computed on the full fitted model and averaged within each sex (Figure A1). Its role in this study is diagnostic rather than predictive: if a flexible learner able to capture arbitrary non-linearities and interactions had recovered a different ordering of determinants in each sex, the null primary result would have required qualification. It did not. The percentage of maximal heart rate (%HRmax) was the dominant predictor in both sexes. In women it was followed by relative intensity, peak oxygen uptake, stature and fat-free mass; in men, stature ranked second, followed by peak oxygen uptake, relative intensity and fat-free mass. The same five predictors therefore occupied the first five positions in both sexes, and no body-composition variable rose to prominence in one sex and not the other.
